# Emerging role of HIC1 in prostate cancer progression and therapeutic response: A novel perspective

**DOI:** 10.1002/ccs3.12032

**Published:** 2025-10-03

**Authors:** Dun Xue, Long Tan, Fengshuai Yang, Xiaolan Tian, Qian Zuo, Xinghui Wu

**Affiliations:** ^1^ Department of Medical The First Hospital of Changsha Changsha China; ^2^ Department of Nurology Surgery The First Hospital of Changsha Changsha China; ^3^ Department of General Surgery The First Hospital of Changsha Changsha China; ^4^ Department of Outpatient Office The First Hospital of Changsha Changsha China; ^5^ Department of Radiology The First Hospital of Changsha Changsha China; ^6^ Department of Urology The First Hospital of Changsha Changsha China

**Keywords:** androgen receptor, castration resistance, HIC1, IRS2, PI3K/AKT axis, prostate cancer

## Abstract

Prostate cancer (PCa) is a leading cause of cancer‐related death in men, with its progression and treatment response being complex. The study focuses on the role of HIC1 (Hypermethylated in cancer 1) in PCa, revealing its downregulation in PCa tissues compared to normal counterparts. Using transcriptome sequencing and bioinformatics, it was found that HIC1 influences key cellular processes like cell growth, proliferation, invasion, and androgen receptor (AR) signaling in PCa. Specifically, AR was identified as a transcription factor for insulin receptor substrate 2 (IRS2), which activates the PI3K/AKT pathway, enhancing PCa cell proliferation and invasion. However, this effect could be reversed by IRS2 inhibition using NT157. Furthermore, HIC1 overexpression reduced castration resistance in PCa cells, with in vivo studies showing that HIC1 silencing increased PCa xenograft growth and resistance, and elevated Ki‐67, Cleaved‐caspase‐3, EMT markers, and prostate‐specific antigen (PSA) levels. Conversely, AR and IRS2 inhibitors like EPI‐7170 and NT157 negatively affected PCa progression. These results underscore HIC1's potential as a therapeutic target in PCa, offering new insights into its role in cancer biology and treatment.

## Graphical abstract

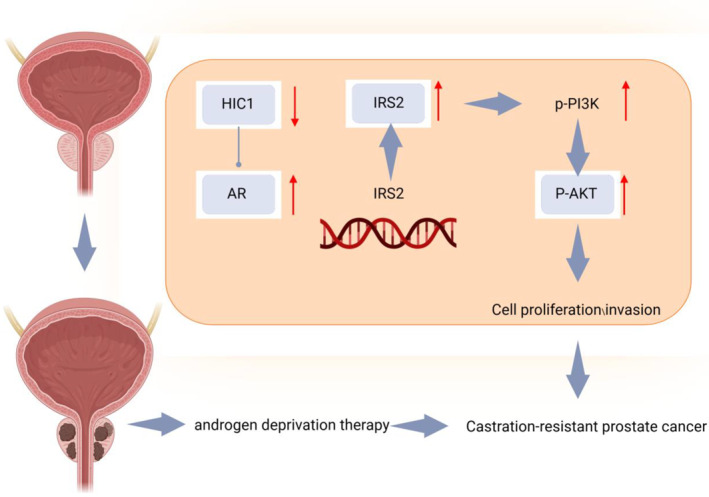

## 1 INTRODUCTION

Prostate cancer (PCa) is a prevalent malignancy that affects men worldwide, particularly in Western countries, where it has the highest incidence and mortality rates compared to other male malignancies.^1^ In recent years, the incidence rate of prostate cancer in China has steadily increased. This rise can be attributed to factors such as an aging population and the adoption of a Western lifestyle.^2^ Surgical removal, radiation therapy, hormone therapy, and chemotherapy are the main treatment modalities for prostate cancer. However, each method has specific side effects and limitations that affect treatment efficacy.^3^, ^4^, ^5^, ^6^ Castration therapy is widely used, but some patients develop castration‐resistant prostate cancer (CRPC) after receiving this treatment, which adds complexity to the therapeutic approach.^7^, ^8^, ^9^

A significant body of research suggests that the androgen receptor (AR) plays a vital role in the development of prostate cancer.^10^, ^11^ The AR is a nuclear receptor that binds to and forms androgen/receptor complexes. These complexes regulate the expression of downstream genes, thereby influencing critical cellular processes, including proliferation, differentiation, and survival.^12^ Elevated expression of the AR is frequently observed in prostate cancer, resulting in increased activity. This enhanced activity allows for continued downstream gene expression even in the presence of lower androgen levels, facilitating the progression of prostate cancer.^13^

Furthermore, the IRS2/PI3K/AKT axis, which involves the insulin receptor substrate 2 (IRS2) signaling pathway, the phosphoinositide 3‐kinase (PI3K), and protein kinase B (AKT), plays a crucial role in prostate cancer development.^14^, ^15^ IRS2 is involved in the insulin/insulin‐like growth factor signaling pathway and promotes cell growth and survival by activating the PI3K/AKT pathway.^16^ Increased expression and activity of IRS2 are commonly observed in various cancers, including prostate cancer, along with enhanced activation of the PI3K/AKT pathway.^17^, ^18^

HIC1, also known as hypermethylated in Cancer 1, is a DNA‐binding protein that regulates critical biological processes, such as cell proliferation, differentiation, and apoptosis. It is closely associated with various types of cancer.^19^ However, the exact role of HIC1 in prostate cancer and its underlying mechanisms remain unclear.^20^, ^21^ This study aims to explore the role of HIC1 in prostate cancer and investigate its potential mechanisms through high‐throughput transcriptome sequencing analysis and cell biology experiments. The objective is to identify new targets and strategies for the treatment of prostate cancer.

## 2 MATERIALS AND METHODS

### 2.1 High‐throughput sequencing of transcriptome samples, conducted quality control of the data, and performed differential gene analysis

Total RNA was extracted from each sample using a Trizol reagent (Thermo, 16096020). The concentration, purity, and integrity of the RNA were measured using the Qubit®2.0 Fluorometer® from Life Technologies (Q33216), the Nanometer spectrophotometer from IMPLEN, and the RNA Nano 6000 Analysis Kit from the Bioanalyzer 2100 system (Agilent, 5067‐1511). These measurements were performed with the Qubit® RNA Analysis Kit from Shanghai Baoji Biotechnology Co. (HKR2106‐01). Each sample contained 3 μg of total RNA and served as the input material for RNA sample preparation. For cDNA library production, the NEBNext® UltraTM RNA Library Prep Kit (NEB, E7435L) designed for use with Illumina® was employed according to the manufacturer's instructions. The quality of the library was subsequently assessed using an Agilent Bioanalyzer 2100 system. The TruSeq PE Cluster Kit v3 cBot HS (Illumina) (PE‐401‐3001) was used to cluster the indexed encoded samples via the cBot cluster generation system, following the manufacturer's instructions. After cluster generation, library preparation was sequenced using the Illumina HiSeq 550 platform, producing 125 bp/150 bp paired‐end reads.^22^, ^23^

To assess the quality of the paired‐end reads in the raw sequencing data, FastQC software version 0.11.8 was used. The raw data were then preprocessed using Cutadapt 1.18 software, which involved removing Illumina sequencing adapters and poly(A) tail sequences, as well as eliminating reads with an N content exceeding 5%, using a Perl script. Reads with a quality score above 20, covering 70% of the bases, were extracted using the FASTX Toolkit version 0.0.13. Paired‐end sequences were repaired using the BBMap software. Finally, the filtered and high‐quality read fragments were aligned to the mouse reference genome using HISAT2 software (version 0.7.12). Differentially expressed genes (DEGs) were selected using the “Limma” package in R, applying a threshold of |logFC| >1.7 and *p*‐value <0.05 for the comparison between Normal and Tumor samples.

For the analysis of differential gene expression, the “heatmap” package and the “ggplot2” package in R were utilized to generate heat maps and volcano plots. Venn diagrams were created using the “vennDiagram” package. Gene ontology (GO) and KEGG pathway enrichment analysis were performed using the “clusterProfiler” package in R, while correlation analysis was conducted using the “corrplot” package. Furthermore, protein‐protein interaction analysis was carried out using the STRING online website.^24^, ^25^

### 2.2 PCa cell culture in vitro

The mouse prostate cancer cell line RM‐1 (catalog number TCM14) was cultured in RPMI 1640 medium containing 10% FBS. The human PCa cell line VCaP (catalog number TCHu220) was cultured in DMEM medium with 20% FBS. PC3 (catalog number TCHu158) was cultured in Ham's F‐12K medium with 10% FBS. LNCaP (catalog number TCHu173) was cultured in RPMI 1640 medium with 10% FBS. DU145 (catalog number TCHu222) was cultured in a DMEM medium with 10% FBS. These cells were all obtained from the Cell Bank of the Chinese Academy of Sciences. HEK‐293 cells were procured from ATCC (catalog number: CRL‐3216, ATCC) and grown in DMEM/F‐12 medium with 10% FBS. The castration‐resistant PCa cell line LNCaP‐AI (LNCaP‐androgen independent) was derived from LNCaP cells after multiple passages, transitioning to a medium containing 10% FBS and charcoal‐stripped serum (CDS medium) devoid of steroids (Israeli Biological Industries), instead of regular FBS. These cells were maintained in a CDS medium for approximately 6 months, resulting in the selection of the castration‐resistant PCa cell line.^26^, ^27^, ^28^, ^29^

For cell culture, a constant‐temperature incubator (catalog number: 51032874, ThermoFisher) was used with 5% CO2 at 37°C. The culture media employed in this study were as follows: FBS (Catalog number: 10099, Gibco), DMEM/F‐12 medium (Catalog number: 21041025, Gibco), DMEM medium (Catalog number: 10566016, Gibco), Ham's F‐12K medium (Catalog number: 21127022, Gibco), and RPMI 1640 medium (Catalog number: 11875093, Gibco). Furthermore, the charcoal‐stripped serum (CSS) used in the study was prepared by treating bovine fetal blood‐derived serum with activated carbon and pectin. The CSS was purchased from Gibco (Catalog number: 12676‐029, USA).

### 2.3 Cell transfection and other treatments

The transfection process involves introducing the pHAGE‐puro series plasmids and helper plasmids pSPAX2 and pMD2.G, as well as the pSuper‐retro‐puro series plasmids and helper plasmids gag/pol and VSVG, into 293t cells. This can be achieved using the Lentivirus Packaging Kit (A35684CN, Invitrogen). When the cells are in the logarithmic phase, they should be digested with trypsin and then seeded into a 6‐well plate at a density of 1 × 10^5^ cells per well. After 24 h of regular culture, when the cell fusion rate reaches approximately 75%, the culture medium should be supplemented with an appropriate amount of packaged lentivirus (MOI = 10, with a working titer of approximately 5 × 10^6^ TU/mL) and 5 μg/mL polybrene (Merck, TR‐1003) for infection. The lentiviral infection should be allowed to proceed for 48 h, during which LNCaP, VCaP, and LNCaP‐AI cells should be treated with 10 μg/mL puromycin (540222, Sigma‐Aldrich) for selection. Puromycin treatment should be maintained for at least 1 week to establish stable transfected cell lines. The titer of the lentivirus is 1 × 10^8^ TU/mL. Please refer to (Table S1) for the specific transfection sequence. All plasmids were acquired from Hanheng Biotech Co., Ltd. in Shanghai, China, at a concentration of 50 ng/mL.

The IRS2 inhibitor NT157 (catalog number M00819, Beijing Bai'aolei Biotechnology Co., Ltd) should be prepared as a 10 mM stock solution according to the provided instructions. For in vitro studies, NT157 should be dissolved in dimethyl sulfoxide (DMSO) to create a 10 mM stock solution and stored at 4°C. For in vivo studies, NT157 should be dissolved in a 20% solution of 2‐hydroxypropyl‐β‐cyclodextrin (2‐HP‐β‐CD) (C0926, Sigma) at a concentration of 5 mg/mL and stored at −80°C. The cells should be treated with a concentration of 5 μM NT157 for 6 h to prepare for further experiments.^30^

Enzalutamide (catalog number MDV3100, MedChemExpress) should be dissolved as instructed and stored at −80°C. The cells should be treated with a concentration of 10 μM enzalutamide for subsequent experiments. To remove the influence of androgens in the culture medium, cells should be cultured in a medium containing 5%–10% FBS‐CSS for 3 days prior to enzalutamide treatment. Subsequently, enzalutamide should be added to the cells in the same medium with 5%–10% FBS‐CSS and incubated for 24 h.^31^

The PI3K inhibitor LY294002 was purchased from Abcam (Catalog number: ab120243). It was dissolved in dimethyl sulfoxide (DMSO) as a 25 mM stock solution according to the manufacturer's instructions and stored at 4°C. Cells were treated with a concentration of 10 μM for 24 h for subsequent experiments.^32^

SC79 was obtained from MedChemExpress (Catalog number: HY‐18749). Enzalutamide was dissolved in DMSO as per the instructions, stored at −80°C, and cells were treated with a concentration of 4 μg/mL for 24 h before subsequent experiments.^33^

The groups for lentiviral infection of cells and drug treatment were as follows: LNCaP cells: sh‐NC, sh‐HIC1‐1, sh‐HIC1‐2, oe‐NC, oe‐AR, oe‐AR+sh‐IRS1, oe‐AR+sh‐IRS2, sh‐HIC1+LY294002 (10 μM), oe‐AR+LY294002 (10 μM), oe‐AR+NT157 (5 μM), Enzalutamide (10 μM), sh‐HIC1+NT157 (5 μM), NC, SC79 (4 μg/mL); VCaP cells: oe‐NC, oe‐HIC1, oe‐HIC1+oe‐AR, sh‐NC, sh‐AR‐1, sh‐AR‐2; LNCaP‐AI cells: oe‐NC, oe‐HIC1, Enzalutamide (10 μM).

### 2.4 Dual luciferase assay

Human embryonic kidney HEK293T cells (iCell‐h237, Cyagen Biosciences) and human prostate cancer cell line (VCaP) were cultured in DMEM medium containing 10% fetal bovine serum at 37°C with 5% CO2. The cDNA fragment of IRS2 containing the AR binding site (IRS2‐WT: GAGGACAGTGGGTAC) was inserted into the pmirGLO vector. Mutant IRS2 cDNA fragment (IRS2‐MUT: GTCCACTCAGGGATG) was synthesized using site‐directed mutagenesis and inserted into the pmirGLO vector. Lipofection was used to co‐transfect HEK293T cells and VCaP cells with recombinant vectors carrying IRS2‐WT or IRS2‐MUT together with oe‐AR or oe‐NC. After transfection, cells were incubated for 48 h and assessed using the Biovision luciferase assay kit (K801‐200). The detection of luciferase reporter gene activity was performed with the Promega Dual‐Luciferase Reporter Assay System (E1910). Renilla luciferase was used as an internal control gene, and the activation level of the target reporter gene was determined by calculating the ratio of firefly luciferase values (RLU) to Renilla luciferase values (RLU).^34^

### 2.5 ChIP

Once the cell fusion reached a level of 70%–80%, formaldehyde was added at a concentration of 1% to fix the cells at room temperature for 10 min. This process was carried out in order to crosslink and stabilize the DNA and proteins within the cells. After crosslinking, the fragmented samples were treated with ultrasound. Each ultrasound treatment lasted for 10 s, with a 10‐s interval between each treatment. This cycle was repeated 15 times to achieve the desired fragmentation of the samples. The samples were then subjected to centrifugation at 12,000 xg at 4°C, and the resulting supernatant was collected and divided into two tubes. The supernatant in each tube was separately incubated overnight at 4°C with two different antibodies: rabbit anti‐IgG (1:100, Abcam, ab172730) as the negative control antibody and anti‐AR antibody (Abcam, ab108341) specific to the target protein. Protein Agarose/Sepharose was used to precipitate the endogenous DNA‐protein complexes. The supernatant was then discarded after a brief centrifugation, and non‐specific complexes were washed. Decrosslinking was performed by overnight incubation at 65°C. Finally, DNA fragments were obtained by extraction and purification using a phenol/chloroform solution. The ChIP‐qPCR products underwent qualitative analysis using 3% agarose gel electrophoresis (Table S2).^35^

### 2.6 Co‐IP validation of HIC1 and AR protein interaction

For immunoprecipitation, the Pierce™ Co‐Immunoprecipitation Kit (26149, Thermo Fisher Scientific, Waltham, MA, USA) was utilized following the manufacturer's instructions. The lysates containing 1 μg of total protein were precleaned with an appropriate homologous IgG antibody (ab6721, 1:2000). Subsequently, the lysates were mixed with 2 mg of anti‐HIC1 or anti‐AR antibodies (Table S3) and gently shaken overnight at 41°C. Protein G agarose (Thermo) was added to each tube, followed by additional gentle shaking at 41°C. The immunocomplexes were washed with cold Radiolabeled Immunoprecipitation Wash Buffer and the selected proteins were eluted from the agarose beads in SDS sample buffer by boiling (0.1 M Tris‐HCl, 10% glycerol, 2% SDS, 0.05% bromophenol blue, and 0.1 M DTT) for 5 min. Similar conditions with IgG antibody (ab6721) were applied to control lanes for each gel, and each sample was resolved on 10% SDS‐PAGE.^36^

### 2.7 RT‐qPCR

Total RNA was extracted from cells and tissues using Trizol (Catalog number: 16096020, purchased from Thermo Fisher Scientific). The concentration and purity of RNA were subsequently measured using Thermo Scientific's NanoDrop One/OneC microvolume nucleic acid and protein concentration meter. The A260/A280 ratio was 2.0, indicating a concentration above 5 μg/μL. For cDNA synthesis, the cDNA first strand synthesis kit (catalog number: D7168L) from Shanghai Beyotime was used. RT‐qPCR experiments were conducted using the RT‐qPCR kit (catalog number: Q511‐02) from Vazyme Biotech in Nanjing, following the instructions in the manual. During the experiment, 2 μL of cDNA template was mixed with 0.2 μL of upstream and downstream primers. This mixture was then added to 10 μL of RT‐qPCR Mix, and the total volume was adjusted to 20 μL with RNase‐free water. PCR amplification was performed using the Bio‐rad CFX96 real‐time quantitative PCR instrument. The reaction conditions comprised pre‐denaturation at 95°C for 30 s, denaturation at 95°C for 10 s, annealing at 60°C for 30 s, and extension at 72°C for 30 s, repeated for 40 cycles. The melting curve range was set between 65°C and 95°C. The primer sequences were designed and provided by Shanghai Bioengineering Co., Ltd. in Shanghai, China. The reference sequence for the primers can be found in Table S4. The relative fold change in gene expression between the experimental and control groups was determined by comparing the ΔΔCt values, with GAPDH serving as the reference gene. The calculation formula is as follows: ΔΔCt = ΔCt in the experimental group − ΔCt in the control group, where ΔCt = Ct (target gene) − Ct (reference gene). The experiment was repeated three times.^37^

### 2.8 Western blot

To extract total proteins from cells and lyse tissues, RIPA lysis buffer (P0013B, Beyotime) containing 1% PMSF was added. The concentration of total protein in each sample was determined using the BCA assay kit (P0011, Beyotime). In order to accommodate the size of the target protein bands, it is recommended to prepare SDS gels with a concentration of 8%–12%. Equal amounts of protein samples were then added into each lane for electrophoretic separation using a pipette. Protein transfer from the gel to a polyvinylidene difluoride (PVDF) membrane (1620177, BIO‐RAD, USA) was followed by blocking with 5% skim milk at room temperature for 1 h. Incubation at 4°C overnight with Table S3 added. Subsequently, the membrane was rinsed three times with 1 × TBST washing buffer at room temperature for 5 min per rinse. Secondary antibodies, specifically goat anti‐rabbit IgG labeled with HRP (ab6721, 1:2000) or goat anti‐mouse IgG labeled with HRP (ab6789, 1:2000), purchased from the UK‐based Abcam company and Cell Signaling Technology, were used and incubated at room temperature for 1 h. The specimens were washed three times with 1 × TBST buffer at room temperature for 5 min each. The gel was then incubated in the ECL reaction solution (1705062, Bio‐Rad), and band exposure imaging was performed using the Image Quant LAS 4000C gel imaging system (GE).^38^ The total cellular protein was normalized using GAPDH as an internal reference. The relative expression levels of proteins were determined by calculating the ratio of grayscale values between the target band and the internal reference band. Protein expression levels were evaluated, and each experimental group was repeated three times.

### 2.9 CCK‐8

The cells were enzymatically dissociated and resuspended, and the cell concentration was adjusted to 1 × 10^3^ cells per well before seeding them in a 96‐well plate for overnight incubation. Cell viability was assessed using the CCK‐8 assay, following the instructions provided by Beyotime in the CCK‐8 kit (C0041). The cells were treated with the CCK‐8 reagent at 24, 48, 72, and 96 h after incubation, and their viability was measured. 10 μL of the CCK‐8 detection solution was added during each measurement, followed by incubation in a cell culture incubator for 1 h. The absorbance at 450 nm was determined using an enzyme‐linked immunosorbent assay (ELISA) reader, and the cell viability was then calculated.^35^

### 2.10 EdU labeling is used to detect cell proliferation rate

The cells were inoculated into a 24‐well plate, with three wells specifically designated for replication within each cell group. To achieve a concentration of 10 μmol/L, EDU (C10310‐1, Guangzhou Ruibo Biotechnology Co., Ltd.) was added to the culture medium. This mixture was then incubated in the incubator for a duration of 2 h. Subsequently, the medium was removed, and the cells were treated with a 4% paraformaldehyde solution in PBS for 15 min. Afterward, the samples were washed twice with phosphate‐buffered saline (PBS) containing 3% bovine serum albumin (BSA). The cells were then incubated at room temperature for 20 min with PBS containing 0.5% Triton‐100 (HFH10, Invitrogen™). Following this, the cells were washed twice with PBS containing 3% BSA. To each well, 100 μl of EDU staining solution was added, and the cells were incubated at room temperature in the dark for 30 min. The nucleus was stained with DAPI (C1002, Biyun Tian Biotechnology Co., Ltd.) for a duration of 5 min. After slide mounting, randomly selected fields of view (ranging from 6 to 10) were observed under a fluorescence microscope, and the number of positive cells present in each field of view was recorded. The EDU labeling index was calculated as the ratio of positive cells to the combined number of positive and negative cells, expressed as a percentage multiplied by 100%.^39^ The experiment was repeated three times on each occasion.

### 2.11 Transwell assay

The in vitro cell invasion assay employed Transwell chambers with an 8 μm pore size (Corning). These chambers consist of a polycarbonate membrane containing Matrigel and are preloaded with 600 mL of culture medium containing fetal bovine serum (FBS) in the lower chamber. After equilibration at 37°C for 1 h, cells were resuspended in FBS‐free DMEM culture medium at a density of 2 × 10^4^ cells/mL and then seeded into the upper chamber. Incubation took place at 37°C with 5% CO_2_ for 24 h. Following incubation, the Transwell chambers were carefully removed and washed twice with PBS. The cells were subsequently fixed with 5% glutaraldehyde at 4°C and stained with 0.1% crystal violet for 5 min. After an additional wash with PBS, surface cells were delicately removed using a cotton ball. The chambers were inverted and examined using a fluorescence microscope (Nikon TE2000). For each group, five random fields were selected and photographed, and the average number of cells that had traversed the chamber was recorded. This experiment was repeated three times independently.^40^

### 2.12 Cloning formation experiment

In cloning experiments, the cells under investigation are initially broken down into single‐cell suspensions. Afterward, 1000 cells were individually placed into culture dishes measuring 6 cm in diameter. To prepare the culture dishes, an appropriate complete medium should be added, and this medium should be replaced every 3 days. Next, the cells are rinsed with PBS and then fixed using 4% paraformaldehyde. Following fixation for 15 min, the cells are stained with 0.4% crystal violet. Colonies containing more than 10 cells are manually counted, and the average count is determined from duplicate wells for statistical analysis.^41^

### 2.13 Experiment to form a sphere

After harvesting and enumerating the cells, they should be transferred to a cell culture plate with a low attachment surface. To each well, 2.5 mL of Complete MammoCult™ Human Culture Medium should be added. The culture dish should then be relocated to a 5% CO_2_, 37°C incubator and incubated for 2–3 days to allow for the observation of tumor spheroid formation. The experiment should be concluded once the diameter of the tumor spheroids exceeds 50 μm. Optical microscopy (Model: SC‐Y100BD, Chensheng Optical Instrument Co., Ltd.) was employed to observe and capture images in the culture plates. Five random fields were selected for counting and subsequent statistical analysis.^42^

### 2.14 PCa mouse orthotopic tumor experiment

Eighty male immunodeficient nude mice (BALB/c, nu/nu), aged 4–5 weeks, were obtained from Beijing Vital River Laboratory Animal Technology Co., Ltd. in Beijing, China. The mice were housed under non‐pathogenic conditions at a temperature of 26–28°C and a humidity range of 50%–65%. All animal experiments were conducted in adherence to ethical guidelines and received approval from our Institutional Animal Ethics Committee.

For sequencing, we implanted two million RM‐1 cells that were fluorescently labeled into the dorsal lobe of the prostate in the mice. D‐fluorescein (150 mg/kg) was injected weekly to monitor tumor growth. After 4 weeks of tumor growth, we collected prostate tissues for sequencing.

For in vivo experiments, we implanted 3 × 10^6^ LNCaP‐sh‐NC or LNCaP‐sh‐HIC1 prostate cell lines that were fluorescently labeled into the dorsal lobe of the mouse prostate. The cell lines were mixed with Matrigel at a 1:1 ratio. D‐fluorescein was administered intraperitoneally at a dose of 150 mg/kg body weight every week to monitor tumor growth. Non‐invasive in vivo bioluminescence imaging was used for imaging. We randomly divided the mice into groups, each consisting of 10 mice. The groups for the castration treatment included LNCaP‐sh‐NC, LNCaP‐sh‐HIC1, LNCaP‐sh‐HIC1 + EPI‐7170, and LNCaP‐sh‐HIC1 + NT157.

The drugs or surgical treatment methods used in this study were as follows: EPI‐7170 treatment involved daily oral administration of EPI‐7170 (30 mg/kg). The EPI‐7170 used in the study was purchased from MedChemExpress (catalog number 150102).^43^ NT157 treatment involved weekly intraperitoneal injections of 50 mg/kg NT157. Castration treatment was performed on the mice once the volume of the transplanted tumor reached approximately 300 mm3 (around 5–6 weeks after transplantation). The inhibitor‐treated group received medication therapy after castration.

Blood samples were obtained weekly from the mouse retro‐orbital sinus using a capillary tube. The collected blood was then centrifuged at 1000 rpm to separate the serum. PSA levels were detected using the PSA ELISA kit (catalog number E‐EL‐M0961c, Elabscience) or through weight measurements. Data collection for a group was ceased if the number of mice fell below 6.^44^

### 2.15 Immunohistochemical experiment

The tissue sample was collected for detection and fixed with formalin, following which paraffin sections measuring 4 μm thick were prepared. Subsequently, the standard immunohistochemical staining methods were employed to deparaffinize and perform the procedure.

In this study, the following antibodies were utilized: Mouse anti‐HIC1 (sc271499; 1:50), Rabbit anti‐Ki67 (ab16667; 1:200), Rabbit anti‐Cleaved caspase‐3 (CST, 9661; 1:400), and Rabbit anti‐Prostate‐specific antigen (ab76113; 1:1000). The antibodies were incubated overnight in a dark room. On a subsequent day, they were incubated with the corresponding secondary antibodies, namely the goat anti‐rabbit IgG antibody (ab6721, 1:1000, Abcam, UK) or goat anti‐mouse IgG antibody (ab6789, 1:1000, Abcam), for a duration of 30 min. Streptavidin‐Biotin Complex (SABC) from Vector Company, USA, was then added and incubated at a temperature of 37°C for 30 min. Drops of the color‐developing reagent from the DAB Color Development Kit (P0203, Beyotime Biotechnology) were applied to the specimen and left to develop for 6 min to facilitate the development of color. Subsequently, the sample was stained with safranin solution for 30 s, followed by sequential immersion in ethanol concentrations of 70%, 80%, 90%, and 95%, and finally, absolute ethanol, with each immersion lasting 2 min, to aid in dehydration. To complete the process, the specimen underwent two rounds of transparent treatment, each lasting 5 min, using xylene. It was then sealed with neutral resin. Finally, observation was carried out using a brightfield microscope (BX63, Olympus, Japan). The staining results will be analyzed utilizing ImageJ software for quantification of grayscale intensity and estimation of protein expression.^45^

### 2.16 Hematoxylin and eosin (H&E) staining

Tumor tissues embedded in paraffin were sectioned at 4 μm, deparaffinized in xylene, and rehydrated through a series of ethanol gradients. The sections were then rinsed in 1 × PBS and stained with hematoxylin and eosin (H&E; Beyotime; C0105). Subsequently, the sections were dehydrated in increasing concentrations of ethanol and xylene, and observed under a bright‐field microscope (BX63, Olympus).^46^

### 2.17 Statistical analysis

Statistical analysis for the bioinformatics findings in our study was carried out using R version 4.2.1, whereas analysis of the remaining results was done with SPSS version 26.0 (IBM). Measurement data is conventionally represented as the mean value ± standard deviation. To begin, tests for normality and homoscedasticity should be conducted. In cases where the data follows a normal distribution and has equal variances, either an independent samples *t*‐test or a paired samples *t*‐test would be appropriate for comparing groups. Statistical significance is denoted by *P* < 0.05.

## 3 RESULTS

### 3.1 Identification and implication of HIC1 downregulation in prostate cancer progression

**FIGURE 1:**
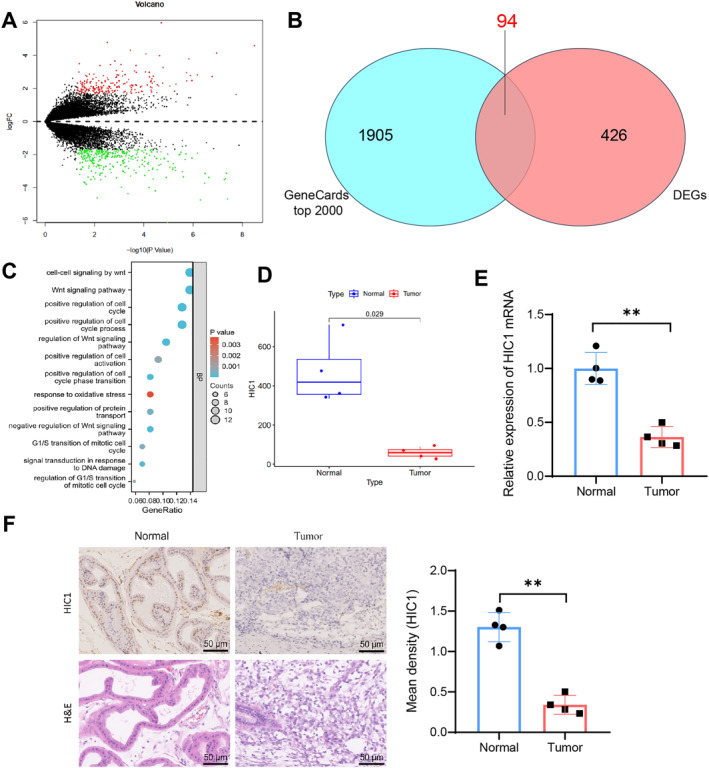
Expression of HIC1 in normal mouse prostate and PCa xenograft prostate tissues. (A) Volcano plot of differentially expressed genes in the Normal group (*n* = 4) and Tumor group (*n* = 4) based on RNA‐seq data, where red represents upregulated genes and green represents downregulated genes; (B) venn diagram showing the intersection of differentially expressed genes in RNA‐seq data and the top 2000 genes associated with prostate cancer according to GeneCards; (C) GO functional enrichment analysis results for 94 intersecting genes shown in the plot; (D) expression of HIC1 in RNA‐seq data; (E) RT‐qPCR analysis of HIC1 mRNA expression in mouse prostate tissues from the Normal and Tumor groups (*n* = 4 each); (F) immunohistochemical analysis of HIC1 expression in mouse prostate tissues from the Normal and Tumor groups (scale bar = 50 μm; *n* = 4 each); *P* < 0.05, *P < 0.01.

Prostate cancer (PCa) is prevalent in Western countries, posing challenges for treatment due to its non‐obvious early symptoms, drug resistance, and complex prognostic factors.^47^ Biomarker testing allows for the early detection and prevention of PCa. In order to investigate the development mechanism of PCa and identify specific biomarkers, we developed a mouse model for PCa tumors through orthotopic transplantation. Using high‐throughput transcriptome profiling via RNA sequencing (RNA‐seq), we analyzed prostate tissues from four normal mice (Normal, *n* = 4) and four PCa model mice (Tumor, *n* = 4). A total of 520 differentially expressed genes (DEGs) were identified in the Tumor group compared to the control Normal group, applying the filtering criteria |LogFC| > 1.7 and *P* < 0.05. Among these DEGs, 199 genes were up‐regulated, and 321 genes were down‐regulated (Figure 1A). Additionally, we obtained the top 2000 genes associated with prostate cancer (PCa) from GeneCards and compared them with the 520 DEGs identified through sequencing, resulting in a set of 94 DEGs specifically associated with PCa, which we refer to as PCa‐DEGs (Figure 1B). The protein‐protein interaction network of these 94 PCa‐DEGs is presented in Figure S1A. A functional enrichment analysis of Gene Ontology (GO) was conducted to explore the relationship between PCa‐DEGs and PCa progression. The results revealed that the 94 PCa‐DEGs were primarily enriched in organelle fission, epithelial cell proliferation, ameboid‐type cell migration, Wnt signaling pathway, and DNA damage (Figure 1C; Figure S1B). Notably, the Wnt signaling pathway and DNA damage are important factors in prostate cancer development.^48^, ^49^ Further, GO enrichment analysis of PCa‐DEGs demonstrated that 12 genes were enriched in the Wnt signaling pathway, and 6 genes were enriched in DNA damage (Figure S1C‐D). Importantly, HIC1 (Hypermethylated in Cancer 1) was found in both gene sets, suggesting its potential strong connection to PCa progression. Previous studies have demonstrated the involvement of HIC1 in the development of various malignant tumors, including breast cancer, lung cancer, and PCa, highlighting its role as a novel tumor suppressor.^50^, ^51^, ^52^, ^53^ However, the regulatory mechanisms of HIC1 in the occurrence and development of PCa have not been thoroughly investigated. To examine the expression of HIC1 in PCa, we analyzed the sequencing expression results of HIC1 (Figure 1D) and further conducted RT‐qPCR and immunohistochemical analysis on PCa xenograft samples obtained from the same batch. The results indicated a decrease in the expression level of HIC1 in PCa (Figure 1D–F). In conclusion, these findings suggest that HIC1 is downregulated in PCa tissue and may be involved in the progression of PCa.

### 3.2 HIC1 inhibits prostate cancer progression by downregulating AR expression and activity

Previous studies have demonstrated that HIC1 enhances the transcriptional activity of the tumor suppressor gene p53 by inhibiting the cellular stress‐responsive protein SIRT1.^54^ In prostate cancer cells, the expression of p53 is decreased, leading to increased expression of the Androgen receptor (AR).^55^ The AR is a critical transcription factor that plays a key role in driving the malignant progression of prostate cancer. Due to its complex mechanisms of action, it has emerged as a pivotal therapeutic target for inhibiting proliferation, metastasis, and the development of castration resistance.^56^, ^57^

**FIGURE 2:**
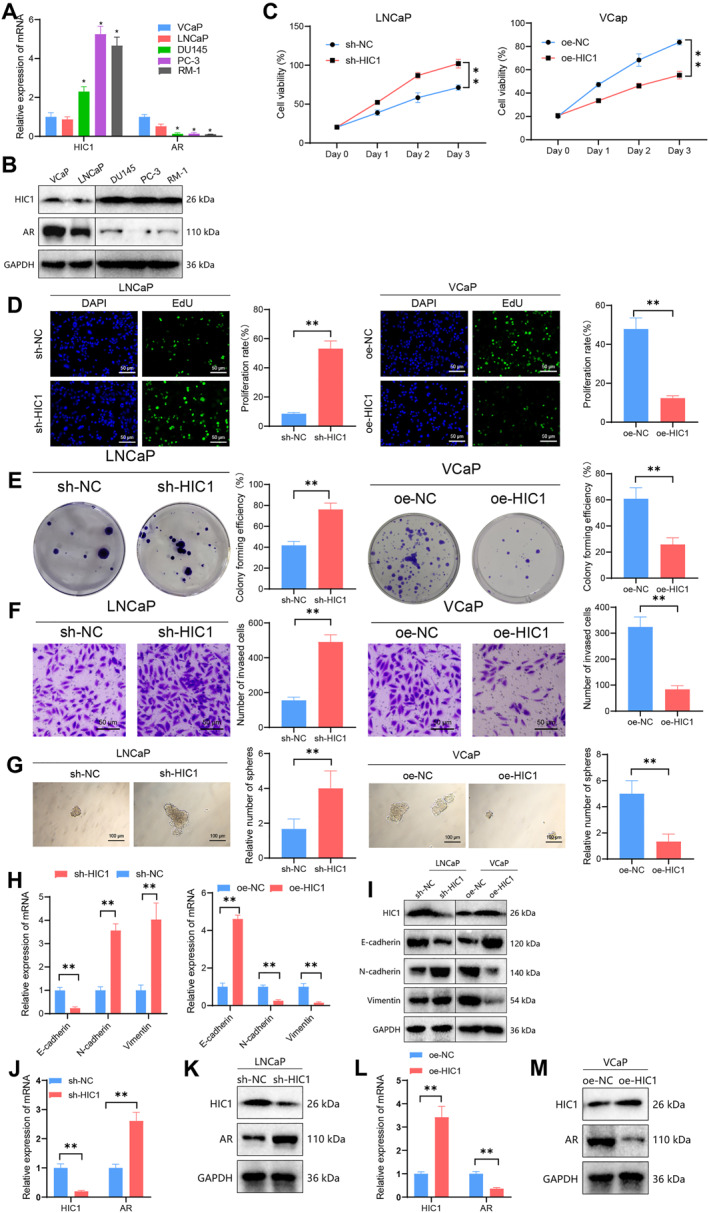
Impact of HIC1 expression on AR expression and cell proliferation and invasion ability in PCa cells. (A and B) mRNA (A) and protein (B) expression of HIC1 and AR in VCaP, PC3, DU145, LNCaP, and RM‐1 PCa cells; (C) CCK‐8 assay to measure the effect of HIC1 silencing and overexpression on cell growth activity; (D) EdU labeling to assess the effect of HIC1 silencing and overexpression on cell proliferation ability (scale bar = 50 μm); (E) clonogenic assay to examine the impact of HIC1 silencing and overexpression on cell proliferation ability; (F) transwell invasion assay to evaluate the effect of HIC1 silencing and overexpression on cell invasion and migration ability (scale bar = 50 μm); (G) sphere formation assay to assess the influence of HIC1 silencing and overexpression on cell self‐renewal and stemness (scale bar = 100 μm); (H and I) RT‐qPCR (H) and Western blot (I) to measure the expression levels of EMT‐related proteins in cells following HIC1 silencing and overexpression; (J and K) mRNA (J) and protein (K) expression of HIC1 and AR in LNCaP cells after HIC1 silencing; (L and M) mRNA (L) and protein (M) expression of AR in VCaP cells after HIC1 overexpression; All cell experiments were repeated three times; **P < 0.01.

To investigate the regulatory role of HIC1 on AR, we initially assessed the expression of HIC1 and AR in human and murine prostate cancer cell lines (including VCaP, PC3, DU145, LNCaP, and RM‐1 cells). Our findings revealed that HIC1 exhibited lower expression levels in VCaP and LNCaP cells, while it was relatively higher in DU145, PC‐3, and RM‐1 cells, contrasting with the expression pattern of AR (Figure 2A,B), indicating a negative correlation between the expression of HIC1 and AR. Consequently, we selected LNCaP and VCaP human prostate cancer cell lines for further investigation.

To assess the effects of HIC1 on prostate cancer cells, we performed lentivirus infection to induce knockdown and overexpression of HIC1 in LNCaP and VCaP cells. The efficacy of knockdown and overexpression was confirmed through RT‐qPCR and Western blot techniques. Our results demonstrated that sh‐HIC1‐1 and sh‐HIC1‐2 effectively knocked down HIC1 (Figure S2A,B), with sh‐HIC1‐2 exhibiting a stronger knockdown effect and thus selected as the preferred candidate (sh‐HIC1). Moreover, overexpression of HIC1 in the oe‐HIC1 group was confirmed (Figure S2C,D), confirming the successful establishment of stable transfectants with HIC1 knockdown in LNCaP cells and HIC1 overexpression in VCaP cells.

Subsequently, we evaluated the effects of HIC1 on the proliferation, migration, invasion, and colony formation of prostate cancer cells using CCK‐8, EdU, invasion experiments, and colony formation assays. Our findings revealed that HIC1 suppression enhanced the growth, proliferation, and invasiveness of prostate cancer cells, while upregulation of HIC1 inhibited these characteristics (Figure 2C–F). Sphere formation experiments further showed that reduction of HIC1 expression increased the self‐renewal and stemness capacities of cells, whereas overexpression of HIC1 diminished these capacities (Figure 2G). Additionally, suppression of HIC1 led to increased expression of Vimentin, a marker protein for epithelial‐mesenchymal transition (EMT), and decreased expression of E‐cadherin. Conversely, overexpression of HIC1 produced the opposite effect (Figure 2H–I), providing further evidence of HIC1's influence on the invasive characteristics of prostate cancer cells.

We then examined the changes in AR expression after intervening in HIC1 expression. Our RT‐qPCR and Western blot analyses demonstrated that downregulation of HIC1 increased AR expression, while overexpression of HIC1 suppressed AR levels (Figure 2J–M). Finally, we overexpressed AR in VCaP cells expressing HIC1 (overexpression confirmed in LNCaP cells, as shown in Figure S2E,F). We observed that AR overexpression in VCaP‐oe‐HIC1 cells stimulated AR expression and enhanced the growth, proliferation, invasive ability, and stemness of prostate cancer cells (Figure S2G–K).

In summary, our results indicate that overexpression of HIC1 decreases AR expression, leading to reduced growth, proliferation, and metastatic potential of prostate cancer cells.

### 3.3 HIC1 regulates prostate cancer progression via the AR/IRS2/PI3K/AKT signaling pathway

In order to investigate the mechanism of HIC1 deletion in the progression of PCa further, we conducted an expression correlation analysis on 94 differentially expressed genes (DEGs) in PCa. Our analysis revealed multiple correlations among these genes (Figure S3A). Based on an extensive review of the relevant literature, we have discovered an association between IRS2 and HIC1. Furthermore, research suggests that IRS2 can potentially function as a transcriptional target gene of the AR.^30^

IRS2 plays a role in tumor development through the PI3K/AKT pathway.^58^ However, the relationship between AR/IRS2 and PI3K/AKT in PCa remains unreported. To investigate this relationship, we conducted a protein‐protein interaction analysis of HIC1, AR, IRS2, PI3K, and AKT using the STRING database, and the results are depicted in Figure S3B. To confirm the association between HIC1 and AR, we performed immunoprecipitation of HIC1 in VCaP cells overexpressing HIC1 (oe‐HIC1) or negative control (oe‐NC). Our findings revealed the presence of both HIC1 and AR in the precipitates. Interestingly, within the precipitates of oe‐HIC1 cells, the protein expression level of AR was lower compared to that in oe‐NC cells (Figure S3C).

**FIGURE 3:**
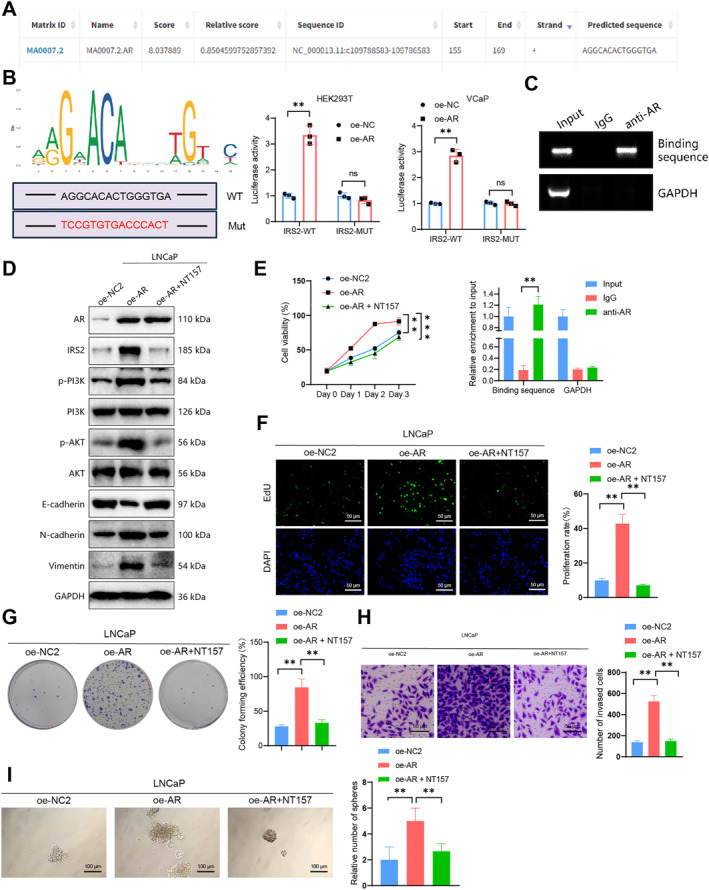
As a transcription factor for IRS2, AR affects the activation of the PI3K/AKT signaling pathway. (A) Prediction of binding sites of AR and IRS2 promoter using JASPAR website; (B) validation of binding sites of AR and IRS2 promoter using a luciferase reporter gene; (C) ChIP assay to verify the interaction of predicted binding sites between AR and IRS2 promoter regions; (D) investigation of the effect of NT157 on IRS2 expression and PI3K/AKT activation in LNCaP cells after AR overexpression; (E) CCK‐8 assay to measure the impact of NT157 on cell proliferation ability in VCaP cells after AR overexpression; (F) EdU labeling to observe the effect of NT157 on cell proliferation ability in VCaP cells after AR overexpression (scale bar = 50 μm); (G) clonogenic assay to evaluate the effect of NT157 on cell proliferation ability in VCaP cells after AR overexpression; (H) transwell assay to measure the effect of NT157 on cell invasion ability in VCaP cells after AR overexpression (scale bar = 100 μm); (I) sphere formation assay to examine the effect of NT157 on cell self‐renewal and stemness in VCaP cells after AR overexpression (scale bar = 25 μm); All cell experiments were repeated three times; ns *P* > 0.05, *P < 0.01, **P < 0.001.

Based on the information above, we propose that HIC1 potentially controls the proliferation and invasion of cancer cells by regulating AR expression and subsequently modulating the IRS2/PI3K/AKT pathway. To further support this hypothesis, we confirmed the transcriptional regulatory function of AR in PCa cells with respect to IRS2. We utilized the JASPAR website to predict the binding sites of AR to the promoter regions of IRS2, as illustrated in Figure 3A. Subsequently, we validated the binding of AR to the predicted site. Results from dual‐luciferase reporter assays in HEK293T and VCaP cells showed a significant increase in luciferase activity in the oe‐AR group compared to the oe‐NC group within the IRS2‐WT co‐transfection group. Conversely, there was no significant difference in luciferase activity between the oe‐NC and oe‐AR groups in the IRS2‐Mut co‐transfection group (Figure 3B).

Furthermore, we conducted chromatin immunoprecipitation (ChIP) experiments using AR and GAPDH antibodies on lysates obtained from VCaP cells, with IgG used as the control. The results revealed the presence of amplified binding sites (Figure 3C), indicating that the AR, acting as a transcription factor for IRS2, can bind to the promoter and regulate the transcription process of IRS2 in PCa.

Moreover, we utilized VCaP cell lines to generate stable transgenic strains in which the AR gene was silenced. The knockdown effect of AR was assessed using RT‐qPCR and Western blot analysis. The results demonstrate a knockdown effect for sh‐AR‐1 and sh‐AR‐2 (Figure S4A,B). We selected the more effective sh‐AR‐2 variant (sh‐AR) for the subsequent experiment. Western blot analysis revealed a downregulation of IRS2 expression after silencing AR, concurrent with a decrease in the phosphorylation levels of PI3K and AKT, indicating the inhibition of PI3K/AKT activation (Figure S4C). NT157 is a selective inhibitor of IRS1/2, which is capable of inducing serine phosphorylation and degradation of IRS1/2.^30^

Treatment with NT157 inhibited the expression levels of IRS2, N‐cadherin, and Vimentin in AR‐overexpressing LNCaP cells (constructed earlier; see Figure 3D). Additionally, NT157 suppressed the activation of the PI3K/AKT pathway. The findings from the EdU labeling, CCK‐8 assay, clone formation assay, invasion assay, and sphere‐forming assay demonstrated that NT157 effectively counteracts the increased proliferation, migration, invasion ability, and enhanced stemness resulting from AR overexpression (Figure 3E–I). To confirm that androgen receptor (AR) acts through IRS2 rather than IRS1, silencing of IRS2 inhibited the overexpression of AR in LNCaP cells and consequently reduced the expression levels of IRS2, N‐cadherin, and Vimentin. Additionally, IRS2 silencing suppressed the activation of the PI3K/AKT pathway upon upregulation of AR, while silencing IRS1 did not lead to any changes in the results (Figure S4D).

Expression levels of IRS2 and the activation status of the PI3K/AKT pathway were subsequently examined by Western blot in cells with silenced or overexpressed HIC1 (Figure S4E). The results indicate that the silencing of HIC1 leads to an increase in IRS2 expression and activates the PI3K/AKT pathway, while the overexpression of HIC1 has the opposite effect. To further substantiate the regulatory role of HIC1 in the AR/IRS2/PI3K/AKT signaling pathway, we employed Western blot analysis to assess the expression of relevant proteins in VCaP cells with HIC1 and AR overexpression. Figure S4F demonstrates that the overexpression of AR reinstated the expression levels of IRS2 and reactivated the PI3K/AKT axis. As shown in Figure S4G, treatment with NT157 inhibited the expression levels of IRS2, N‐cadherin, and Vimentin in HIC1 knockdown LNCaP cells, concomitantly suppressing the activation of the PI3K/AKT pathway. To further confirm the impact of the PI3K/AKT signaling pathway on LNCaP cell proliferation, we introduced a PI3K inhibitor (LY294002) in sh‐HIC1 or oe‐AR cells. The results demonstrated that the addition of LY294002 in sh‐HIC1 cells inhibited the activation of the PI3K/AKT pathway induced by HIC1 knockdown, as indicated in Figure S5A, and also suppressed the growth of prostate cancer cells, shown in Figure S5C,D. Consistent results were obtained when LY294002 was added to oe‐AR cells, as shown in Figure S5B, S5E,F. Furthermore, as depicted in Figure S5G–I, the activation of the AKT pathway was promoted upon treatment with an AKT activator (SC79) in the LNCaP cell line, leading to enhanced cell proliferation.

Our findings suggest that the AR functions as a transcription factor for IRS2 downstream of HIC1. This process promotes the expression levels of IRS2 and activates the PI3K/AKT signaling axis, ultimately enhancing the growth and invasive potential of PCa cells.

### 3.4 HIC1 overexpression inhibits castration resistance and attenuates aggressive behavior in PCa cells

**FIGURE 4:**
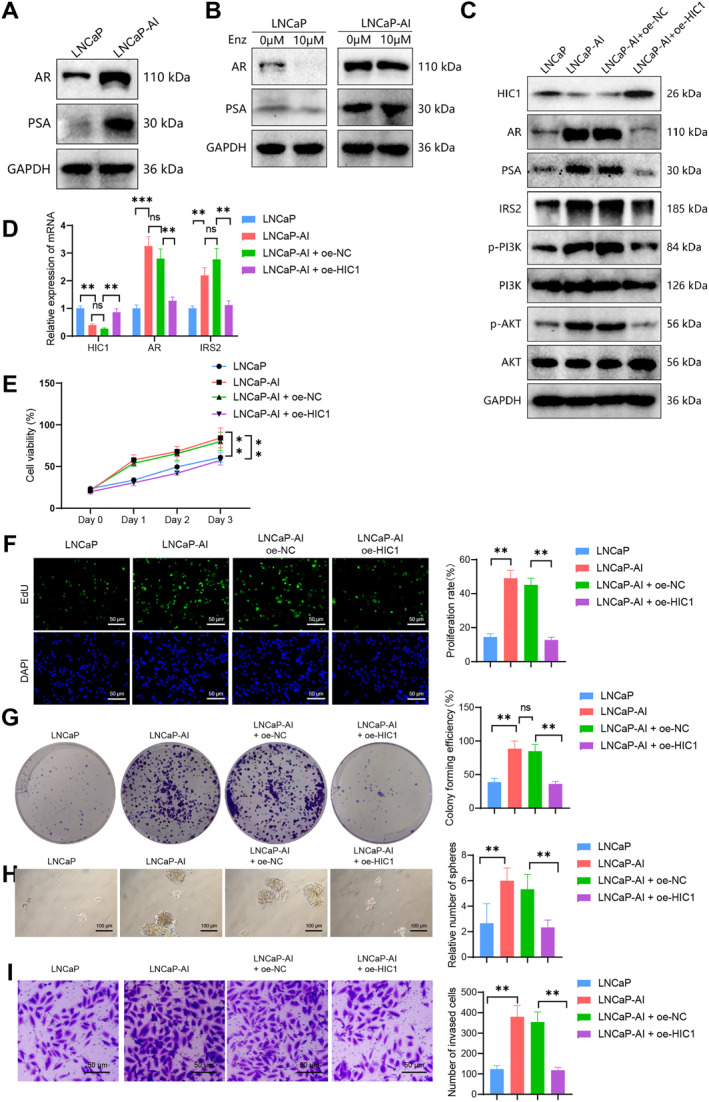
The impact of HIC1/AR/IRS2 on the development of castration resistance in PCa cells. (A) Western blot analysis of AR and PSA expression levels in cells; (B) assessment of cell sensitivity to Enzalutamide treatment as an AR antagonist; (C) western blot analysis of AR/IRS2/PI3K/AKT activation and detection of castration resistance through PSA expression; (D) RT‐qPCR analysis of AR/IRS2 mRNA expression levels in cells; (E) CCK‐8 assay to measure cell growth activity after HIC1 overexpression; (F) EdU labeling to evaluate cell proliferation ability after HIC1 overexpression (scale bar = 25 μm); (G) clonogenic assay to assess cell proliferation ability after HIC1 overexpression; (H) sphere formation assay to measure cell self‐renewal and stemness after HIC1 overexpression (scale bar = 25 μm); (I) transwell invasion assay to analyze cell invasion and migration ability after HIC1 overexpression (scale bar = 50 μm); All cell experiments were repeated three times; nsP > 0.05, **P < 0.01.

The development of PCa into castration resistance represents the culmination of clinical treatment, as cancer cells persist in their growth and metastasis, ultimately leading to reduced patient survival.^59^ In this study, we aimed to investigate the impact of HIC1 on the development of castration resistance in PCa cells. To achieve this, we established a castration‐resistant PCa cell model called LNCaP‐AI using LNCaP cells. The detailed methodology can be found in the literature. We assessed the levels of androgen receptor (AR) and prostate‐specific antigen (PSA) expression in the model to confirm the successful establishment of our experimental model. Additionally, we evaluated the responsiveness of AR to validate the model further (Figure 4A,B). The results demonstrated higher expressions of AR and PSA in LNCaP‐AI cells compared to LNCaP cells.

Subsequent treatment experiments were conducted using the AR antagonist Enzalutamide. The results indicated that LNCaP‐AI exhibited no response to the treatment, confirming the successful establishment of our castration‐resistant cell model, LNCaP‐AI. Notably, castration resistance is characterized by androgen‐independent and hormone‐refractory features. Overexpression of the HIC1 gene weakened the castration resistance of LNCaP‐AI cells, leading to a significant decrease in AR and PSA expression levels. Additionally, there was a reduction in the activation of the IRS2/PI3K/AKT signaling pathway (Figure 4C,D).

Moreover, the results obtained from the CCK‐8, EdU assay, flat plate cloning assay, sphere formation assay, and Transwell assay collectively demonstrated a decrease in the proliferative, invasive, and stem cell‐like properties of LNCaP‐AI cells with HIC1 overexpression (Figure 4E–I).

In conclusion, our research suggests that HIC1 may have the potential to suppress the development of castration‐resistant PCa cells.

### 3.5 HIC1 modulates prostate cancer growth, metastasis, and castration resistance via the AR/IRS2/PI3K/AKT axis in vivo mouse models

**FIGURE 5:**
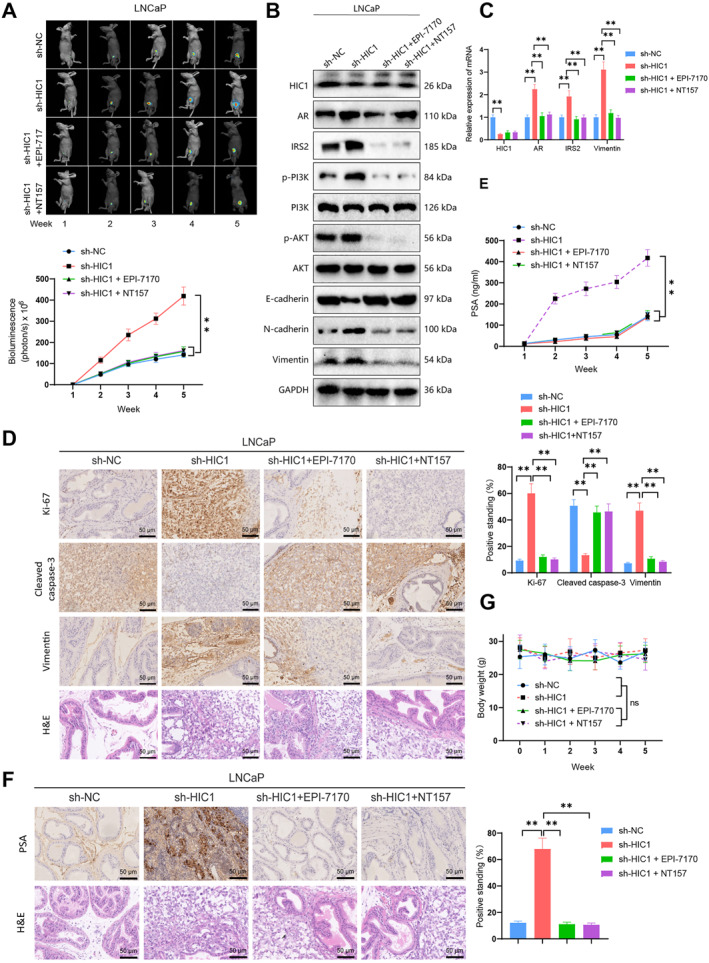
Impact of HIC1 on PCa tumor growth through the AR/IRS2 axis. (A) Observation of tumor growth in LNCaP‐sh‐HIC1 and EPI‐7170, NT157‐treated mice with orthotopic prostate xenografts; (B and C) western blot (B) and RT‐qPCR (C) to measure protein and RNA expression levels of relevant molecules in LNCaP‐sh‐HIC1 and EPI‐7170, NT157‐treated orthotopic prostate xenografts; (D) immunohistochemical analysis of Ki‐67, Cleaved‐caspase‐3, and Vimentin expression in prostate tissues of LNCaP‐sh‐HIC1 and EPI‐7170, NT157‐treated orthotopic prostate xenografts (scale bar = 100 μm); (E) quantification of PSA in serum of LNCaP‐sh‐HIC1 and EPI‐7170, NT157‐treated orthotopic prostate xenograft models; (F) immunohistochemical evaluation of PSA expression in LNCaP‐sh‐HIC1 and EPI‐7170, NT157‐treated orthotopic prostate xenografts (scale bar = 50 μm); (G) observations of body weight changes in LNCaP‐sh‐HIC1 and EPI‐7170, NT157‐treated mice (*n* = 10 each); nsP > 0.05, **P < 0.01.

This study utilized an in vivo mouse model to comprehensively investigate the impact of HIC1 on the proliferation, invasion, and development of castration resistance in PCa. We injected fluorescently labeled HIC1 silenced (LNCaP‐sh‐HIC1) cell line and control group (LNCaP‐sh‐NC) into the prostate lobe and administered the treatment according to the described methods. This included orally administering the AR inhibitor EPI‐7170 (at a daily dose of 30 mg/kg) and intraperitoneally administering the IRS2 inhibitor NCT157 (at a dose of 50 mg/kg, three times weekly). Tumor growth was assessed through palpation every other day and monitored weekly using non‐invasive in vivo bioluminescence imaging. Mice injected with PCa cells exhibited a 100% tumor incidence. Tumors in the group with low HIC1 expression showed accelerated growth, whereas tumors in the groups treated with EPI‐7170 and NT157 displayed slower growth after HIC1 silencing (Figure 5A).

In this study, we evaluated the expression levels of HIC1, AR, IRS2, and EMT‐related proteins, as well as the activation status of the PI3K/AKT pathway in PCa mouse tumor tissue using RT‐qPCR and Western blot experiments. Figure 5B,C provides evidence for the results. The findings suggest that suppressing HIC1 in PCa mouse tissues leads to increased expression of AR and IRS2 while decreasing the expression of E‐cadherin, a protein associated with epithelial‐mesenchymal transition (EMT). Furthermore, it resulted in elevated levels of N‐cadherin, Vimentin, and activation of the PI3K/AKT pathway. Treatment with EPI‐7170 and NT157 reversed these effects.

To further investigate the impact of HIC1/AR/IRS2 on the proliferation and metastasis of PCa cells, we conducted immunohistochemical analysis on PCa xenograft tumor slices. This analysis included markers for cell proliferation (Ki‐67), apoptosis (Cleaved caspase‐3), and epithelial‐to‐mesenchymal transition (Vimentin). As shown in Figure 5D, PCa cells silenced for HIC1 exhibited increased expression of Ki67 and Vimentin, while the expression of Cleaved caspase‐3 decreased. Conversely, the PCa tissues in the EPI‐7170 and NT157 treatment groups displayed contrasting outcomes.

The serum level of Prostate‐Specific Antigen (PSA) can serve as an indicator for the recurrence of PCa. During our weekly monitoring of PSA levels in mice with PCa, we observed that as the tumor grew, PSA levels increased. However, the administration of EPI‐7170 and NT157 decreased PSA levels in mice with low HIC1 expression, as shown in Figure 5E. Immunohistochemistry analysis of mouse tumor tissue also confirmed these changes in PSA levels, as depicted in Figure 5F. Throughout this study, no significant effects of inhibitor treatment on the body weight of mice were observed (Figure 5G).

Interestingly, the overexpression of HIC1 had the opposite effect. It decreased the expression of the AR/IRS2/p‐PI3K/p‐AKT axis in prostate tissue, inhibited the growth of PCa xenograft tumors, and reduced the level of PSA expression (Figure S6A–E). This finding suggests that HIC1 can suppress the proliferation and epithelial‐mesenchymal transition (EMT) of PCa cells by inhibiting AR/IRS2. Additionally, it promotes apoptosis, ultimately leading to the inhibition of tumor growth.

**FIGURE 6:**
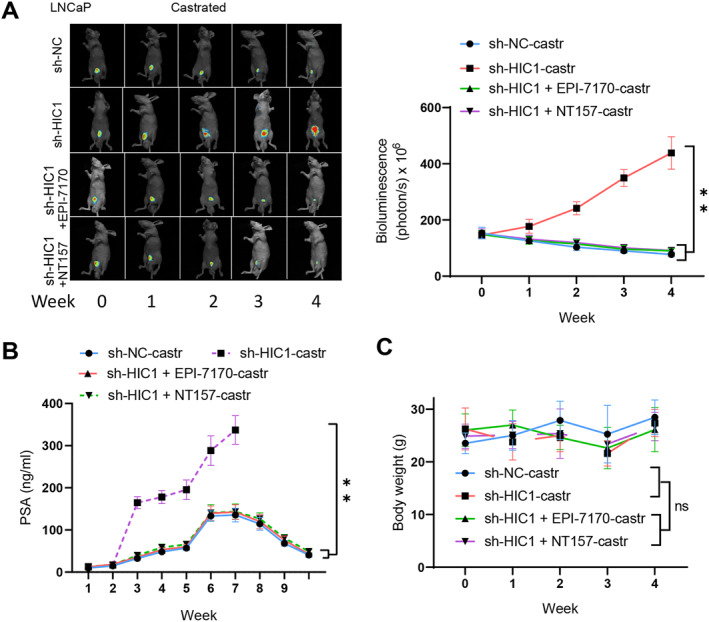
HIC1 influences castration resistance in PCa tumors through the AR/IRS2 axis. (A) Tumor growth of LNCaP‐sh‐HIC1, EPI‐7170, and NT157‐treated xenografts after castration resistance therapy in mice. (B) serum PSA levels of LNCaP‐sh‐HIC1, EPI‐7170, and NT157‐treated xenografts after castration resistance therapy in mice. (C) changes in body weight of LNCaP‐sh‐HIC1, EPI‐7170, and NT157‐treated xenograft mice after castration resistance therapy. (*n* = 10 per group); nsP > 0.05, **P < 0.01.

To examine the impact of HIC1/AR/IRS2 on the progression of castration‐resistant prostate cancer, we injected HIC1‐silenced cells (LNCaP‐sh‐HIC1) and a control group (LNCaP‐sh‐NC) labeled with fluorescence into the posterior lobe of the prostate. When the tumor growth in the low‐expression HIC1 group reached 300 mm3, the mice were castrated and randomly assigned to either the EPI‐7170 or NT157 group. EPI‐7170 was administered orally once daily at a dose of 30 mg/kg, while NT157 was administered via intraperitoneal injection three times a week at a dose of 50 mg/kg. Tumor growth was monitored using non‐invasive in vivo bioluminescence imaging. The results demonstrate that castration treatment was ineffective in the low‐expression HIC1 group compared to the sh‐NC group, whereas the average tumor volume decreased in the EPI‐7170 and NT157 treatment groups (Figure 6A). The expression level of PSA correlated with the condition of tumor growth, as shown in Figure 6B. No weight effects of NT157 on animals were observed throughout the treatment period (Figure 6C).

In conclusion, HIC1 plays a critical role in suppressing the proliferation, metastasis, and castration resistance of prostate cancer cells in an in situ mouse model. It achieves this by influencing the regulation of the AR on the IRS2/PI3K/AKT signaling axis.

## 4 DISCUSSION

This study employed high‐throughput transcriptome sequencing analysis to identify a decrease in the expression of the tumor suppressor HIC1 in prostate cancer cells. These findings align with previous research, which has emphasized the inhibitory role of HIC1 in various tumor types.^19^, ^60^ HIC1 functions as a transcription factor responsible for encoding ribosomal proteins, with prior studies confirming its essential role in DNA damage response, cell cycle regulation, and maintenance of genomic stability, all critical for normal cellular function.^61^ This study sheds light on the involvement of HIC1 in prostate cancer and proposes that the regulation of the AR/IRS2 axis represents a potential mechanism through which HIC1 impacts the development of prostate cancer.

The outcomes of the experiments conducted on prostate cancer cells demonstrate the influence of HIC1 expression on cell growth, proliferation, invasion, and stemness, aligning with its role in other types of cancer.^20^ Our constructed cell models, wherein HIC1 was either overexpressed or silenced, clearly demonstrated the crucial role of HIC1 in controlling the proliferation and invasion of prostate cancer cells. These findings provide new insights into the mechanisms underlying HIC1's action. This study reveals that HIC1 inhibits the progression of prostate cancer by regulating the AR/IRS2 axis, thus addressing a research gap and enhancing our understanding of HIC1's mechanism of action. The JASPAR database predicted binding sites of AR and IRS2 promoters, and these predictions were experimentally validated to confirm the interaction between HIC1, AR, and IRS2.

Furthermore, the inhibition of IRS2 by NT157 effectively counteracted the enhanced cell proliferation and invasive capacity resulting from AR overexpression and activation of the PI3K/AKT pathway. These findings offer promising targets for the development of drugs for prostate cancer treatment in the future. Castration therapy is a frequently employed treatment for prostate cancer, but as treatment advances, patients may develop castration resistance, posing challenges in clinical practice.^62^, ^63^, ^64^ Our research findings indicate that the overexpression of HIC1 plays a crucial role in reducing castration resistance in prostate cancer cells. This discovery significantly enhances our understanding and potential treatment strategies for castration‐resistant prostate cancer.

We created a mouse model of prostate cancer and observed that silencing HIC1 increased the expression levels of Ki‐67, Cleaved‐caspase‐3, and EMT‐related proteins in prostate cancer cells. Additionally, the levels of PSA in serum and tissue were also elevated. These in vivo experiments further strengthen the evidence supporting the role of HIC1 in prostate cancer progression.

**FIGURE 7:**
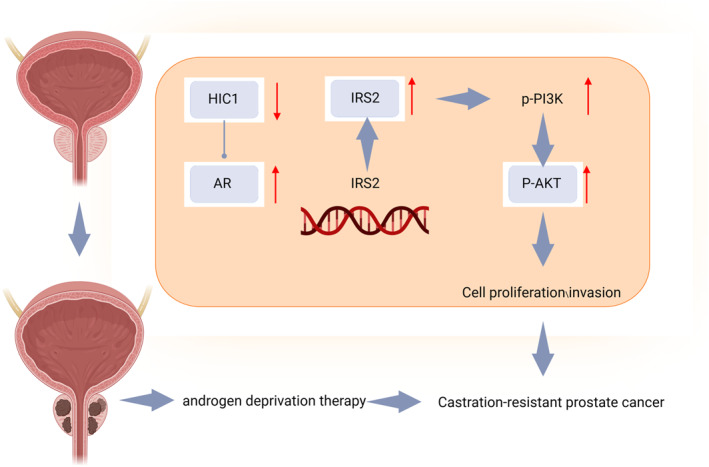
HIC1 inhibits AR expression to suppress the development of castration‐resistant prostate cancer through the IRS2/PI3K/AKT axis.

Based on the aforementioned experimental results, several preliminary conclusions can be drawn. Firstly, the expression of HIC1 in PCa tissues is decreased compared to normal prostate tissue, resulting in an upregulation of AR levels. Moreover, the overexpression of AR promotes the transcription of IRS2, subsequently activating the PI3K/AKT signaling pathway and promoting the progression of PCa. Additionally, our findings indicate a strong correlation between HIC1 and resistance to castration therapy in PCa. We observed the inhibition of castration‐resistant PCa progression in vitro and in vivo following HIC1 overexpression. This inhibitory mechanism is mediated by the AR/IRS2/PI3K/AKT signaling pathway (Figure 7). The study contributes to scientific knowledge by thoroughly exploring the pathogenesis of PCa. Our study utilized high‐throughput transcriptome sequencing analysis to comprehensively elucidate the role of HIC1, a novel tumor suppressor, in the pathogenesis of prostate cancer. These findings advance our understanding of the mechanistic basis of PCa onset and progression. Furthermore, our study investigated how HIC1 controls the AR/IRS2 axis to inhibit the proliferation, invasion, and castration resistance in PCa. This research lays a theoretical foundation for furthering the comprehension of molecular therapy approaches for PCa.

Firstly, the downregulated expression of HIC1 can serve as a biomarker for prostate cancer, enhancing the early detection rate of the disease. Moreover, the inhibitory effects of HIC1 on PCa cell proliferation and invasion, along with its potential to decrease castration resistance, suggest that HIC1 could be a promising therapeutic target for prostate cancer, opening up new avenues for clinical treatment. Our comprehensive study on the AR/IRS2/PI3K/AKT axis also provides theoretical support for the development of more effective treatment strategies for prostate cancer.

Although our research has yielded some significant achievements, certain limitations still need to be addressed. Firstly, our experiments primarily utilized mouse models, and further studies are necessary to validate the complete applicability of the results to human conditions. Furthermore, while our research has demonstrated the potential of HIC1 in inhibiting the AR/IRS2 axis and suppressing PCa proliferation, invasion, and castration resistance, the precise mechanism by which HIC1 regulates this axis and the detailed molecular processes involved remain to be fully understood. Therefore, further investigation is necessary. Additionally, more research is needed to explore HIC1 as a potential therapeutic target, including evaluating its efficacy, safety, and potential for drug resistance.

Our research has provided novel insights into the pathogenesis and clinical management of prostate cancer. In the future, we aim to delve deeper into the specific molecular mechanisms by which HIC1 inhibits the AR/IRS2 axis, enabling us to identify more precise intervention points and enhance the targetedness and effectiveness of treatment. Moreover, we aim to examine the viability of HIC1 as a biomarker by conducting extensive clinical trials to enhance early detection rates for prostate cancer. Lastly, we plan to conduct clinical trials to assess the effectiveness and safety of a new treatment approach targeting HIC1, offering improved treatment alternatives for patients with prostate cancer.

## AUTHOR CONTRIBUTIONS

**Dun Xue**: Conceived and designed the experiments; Contributed reagents, materials, analysis tools or data. **Long Tan**: Performed the experiments; Analyzed and interpreted the data. **Fengshuai Yang**: Contributed to the writing of the manuscript; Prepared figures and/or tables. **Xiaolan Tian**: Oversaw the research project; Contributed to the writing and critical revision of the manuscript. **Qian Zuo**: Conducted the field work; Collected data, and contributed to the manuscript's discussion and conclusions. **Xinghui Wu**: Contributed to the revision process of the manuscript.

## CONFLICT OF INTEREST STATEMENT

The author declares no conflict of interest.

## ETHICS STATEMENT

All experiments involving mice were approved by the Animal Ethics Committee of the First Hospital of Changsha.

## Supporting information

Supporting Information S1

Figure S1

Figure S2

Figure S3

Figure S4

Figure S5

Figure S6

## Data Availability

The datasets generated and/or analyzed during the current study are available from the corresponding author on reasonable request.
