# Supplementary material for: Emerging role of HIC1 in prostate cancer progression and therapeutic response: A novel perspective
Source: J Cell Commun Signal. 2025 Oct 3;19(4):e12032. doi: 10.1002/ccs3.12032 (PMC12494493; doi:10.1002/ccs3.12032)
Supplement: Supplementary file 1 — Supporting Information S1 [file CCS3-19-e12032-s006.docx]

**Table S1. Silent lentivirus sequence**

| Name | Sequence（5’-3’） |
| --- | --- |
| sh-NC | CCTAAGGTTAAGTCGCCCTCG |
| sh-HIC1-1 | TGTGCAAGAAACGCCTCAA |
| sh-HIC1-2  sh-AR-1  sh-AR-2  sh-IRS1  sh-IRS2 | TGTGCAAGAAACGCCTCAA  GCAGAAATGATTGCACTATTG  GCTGACAGTGTCACACATTGA  GCCGCTCAAGTGAGGATTTAA  GCTGGATGAATACACCCTGAT |

**Table S2. ChiIP qPCR sequence**

| Gene | Primer sequence（5’-3’） |
| --- | --- |
| P1（Human） | F: GGTGCACAGACTTAAAATGGC  R: TGGGAAGAATAATCATTGTGGCA |

Notes：F, forward；R, reverse

**Table S3. Antibody manufacturer information**

| Name（Human） | Cat.No | Dilution ratio | Manufactor | Country |
| --- | --- | --- | --- | --- |
| Murine antibody HIC1 | sc271499 | 1:500 | Santa Cruz | CA |
| Rabbit anti AR | 3202S | 1:500 | CST | US |
| Rabbit anti IRS1 | 2382S | 1:1000 | CST | US |
| Rabbit anti IRS2 | ab134101 | 1:2000 | Abcam | UK |
| Rabbit anti p-PI3K | ab182651 | 1:1000 | Abcam | UK |
| Rabbit anti PI3K | ab302958 | 1:2000 | Abcam | UK |
| Rabbit anti p-AKT | ab38449 | 1:1000 | Abcam | UK |
| Rabbit anti AKT | ab8805 | 1:1000 | Abcam | UK |
| Rabbit anti N-cadherin | ab207608 | 1:1000 | Abcam | UK |
| Rabbit anti E-cadherin | ab40772 | 1:10000 | Abcam | UK |
| Rabbit anti Vimentin | ab92547 | 1:2000 | Abcam | UK |
| Rabbit anti prostate specific antigen | ab76113 | 1:1000 | Abcam | UK |
| Rabbit anti GAPDH | ab181602 | 1:10000 | Abcam | UK |

**Table S4. RT-qPCR sequence**

| Gene | Primer sequenc（5’-3’） |
| --- | --- |
| HIC1（Human） | F: GTCGTGCGACAAGAGCTACAA  R: CGTTGCTGTGCGAACTTGC |
| HIC1（Mouse） | F: AACCTGCTAAACCTGGACCAT |
|  | R: CCACGAGGTCAGGGATCTG |
| AR（Human） | F: CCAGGGACCATGTTTTGCC |
|  | R: CGAAGACGACAAGATGGACAA |
| IRS2（Human） | F: CGGTGAGTTCTACGGGTACAT |
|  | R: TCAGGGTGTATTCATCCAGCG |
| Vimentin（Human） | F: AGTCCACTGAGTACCGGAGAC |
|  | R: CATTTCACGCATCTGGCGTTC |
| GAPDH（Human） | F: AATGGGCAGCCGTTAGGAAA  R: GCGCCCAATACGACCAAATC |

Notes：F, forward；R, reverse
