# Supplementary figures and images for: Emerging role of HIC1 in prostate cancer progression and therapeutic response: A novel perspective
Source: J Cell Commun Signal. 2025 Oct 3;19(4):e12032. doi: 10.1002/ccs3.12032 (PMC12494493; doi:10.1002/ccs3.12032)

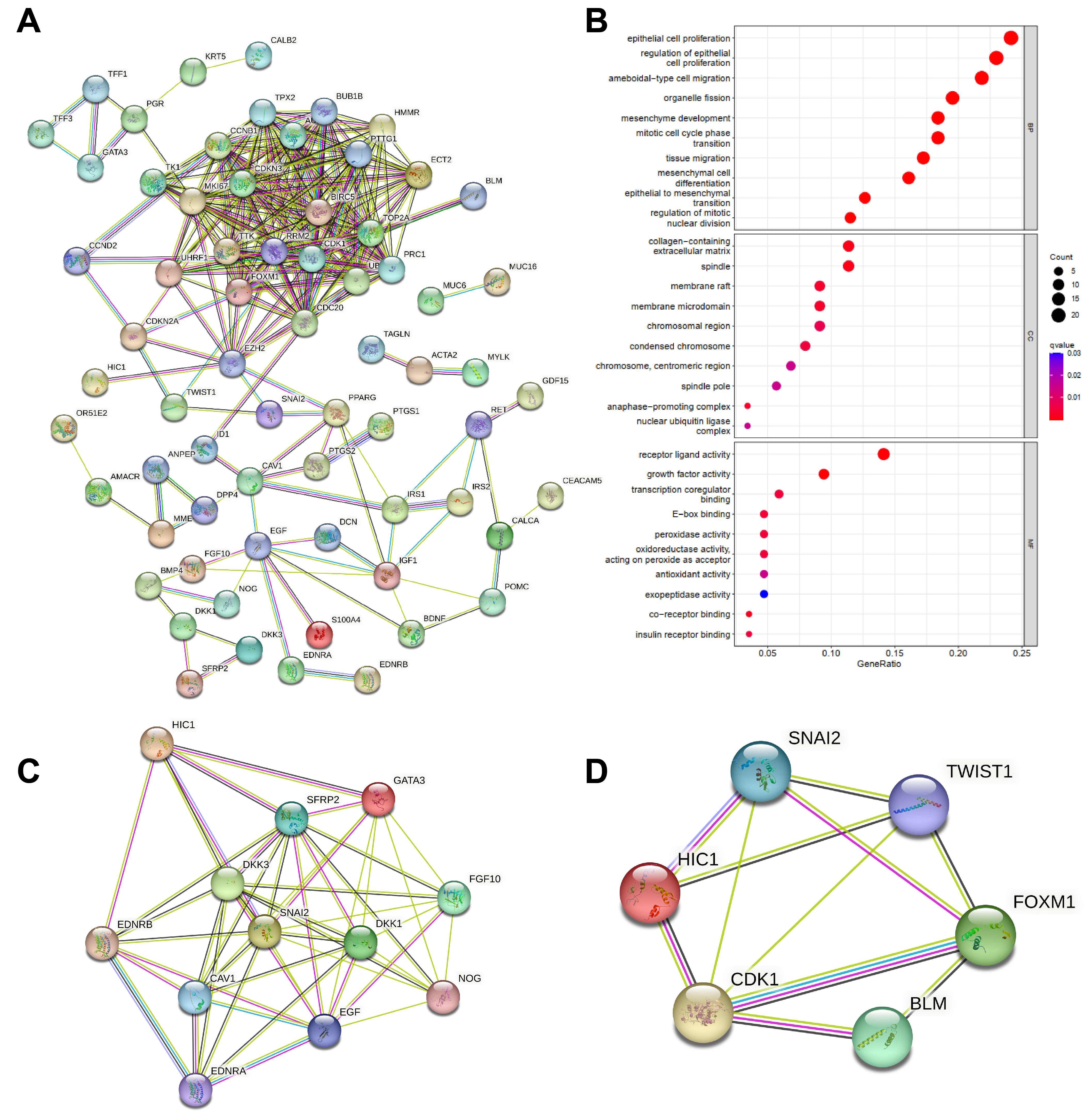

Supplement: Supplementary file 2 — Figure S1 [file CCS3-19-e12032-s001.jpg]

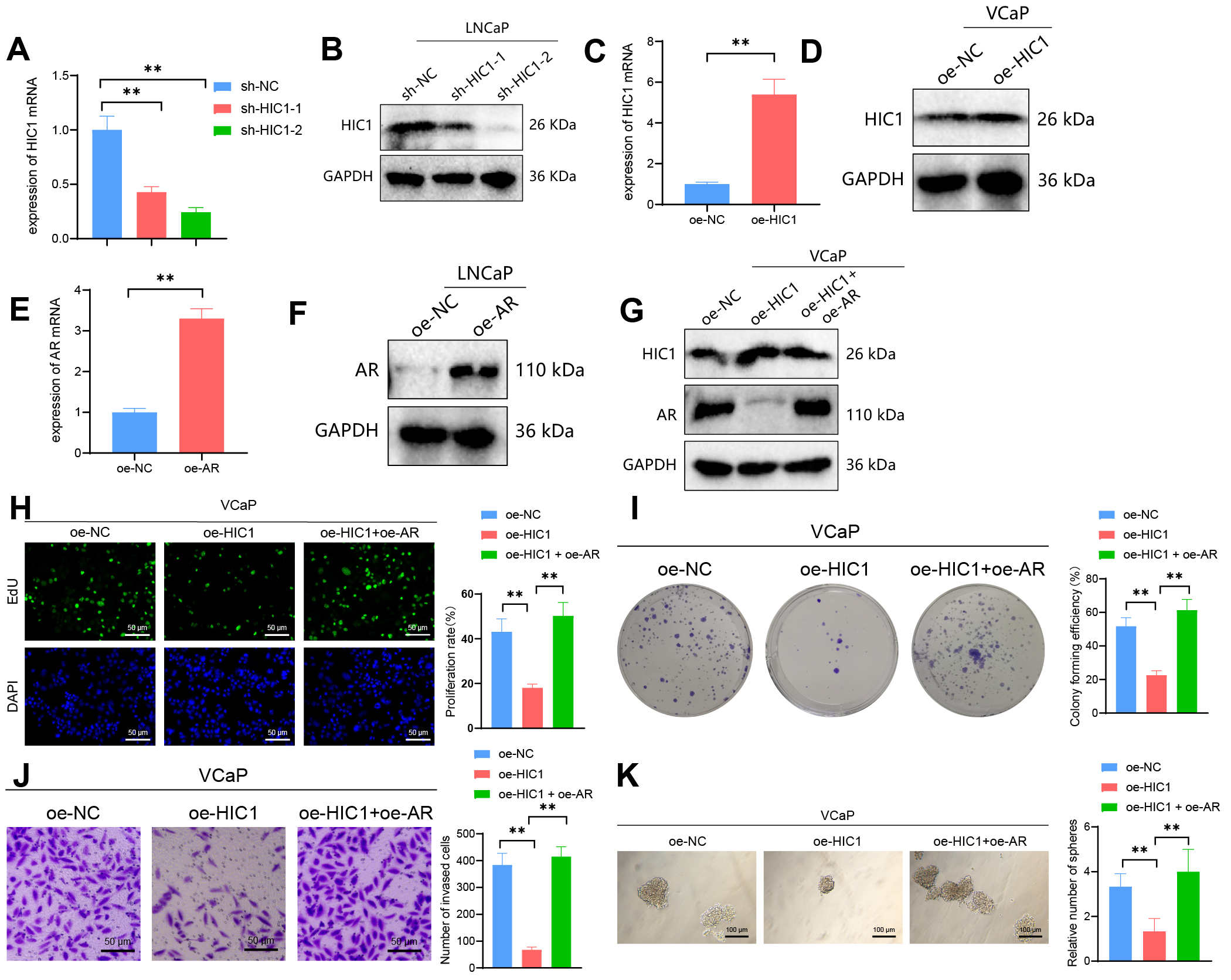

Supplement: Supplementary file 3 — Figure S2 [file CCS3-19-e12032-s003.jpg]

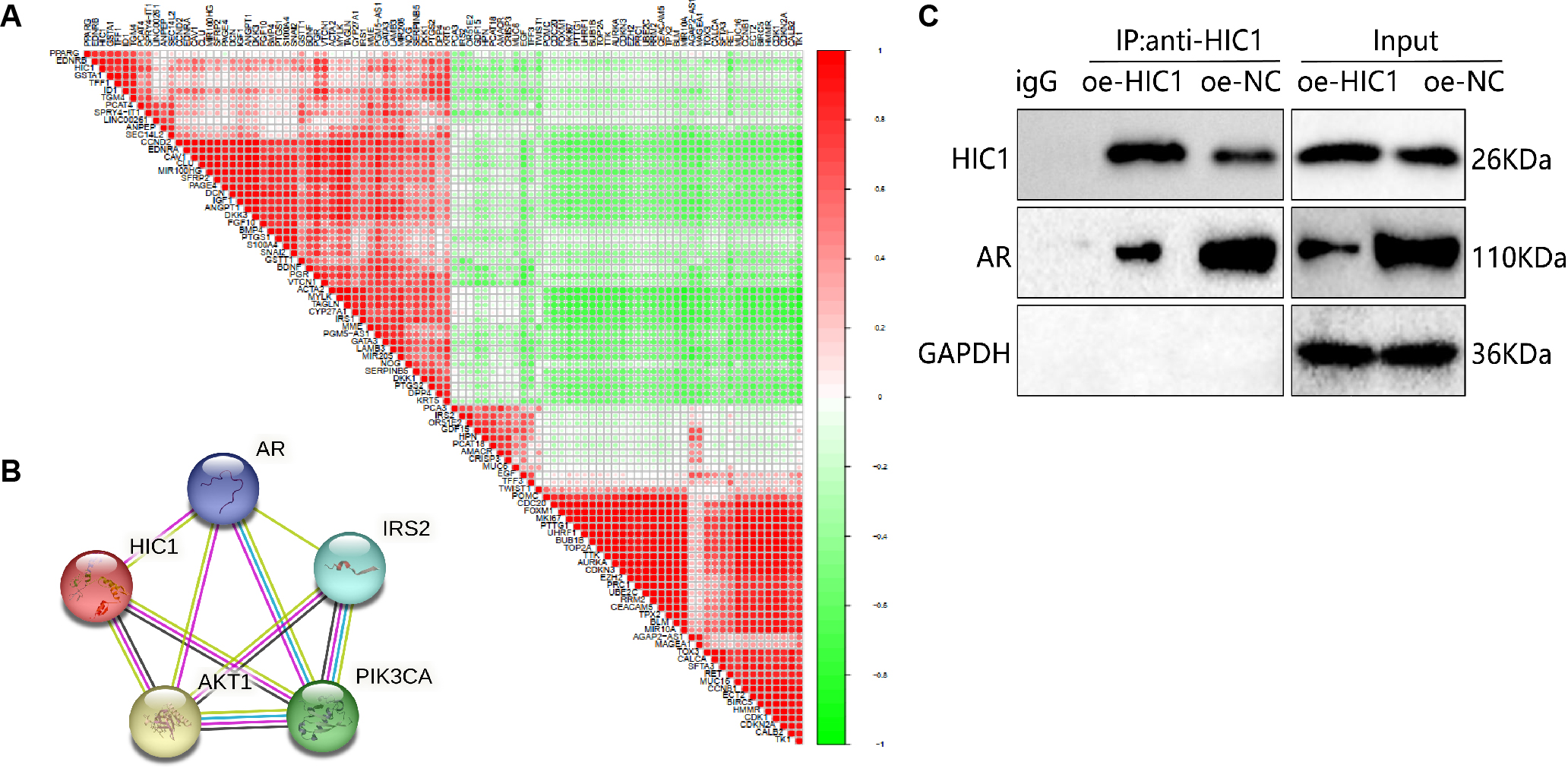

Supplement: Supplementary file 4 — Figure S3 [file CCS3-19-e12032-s005.jpg]

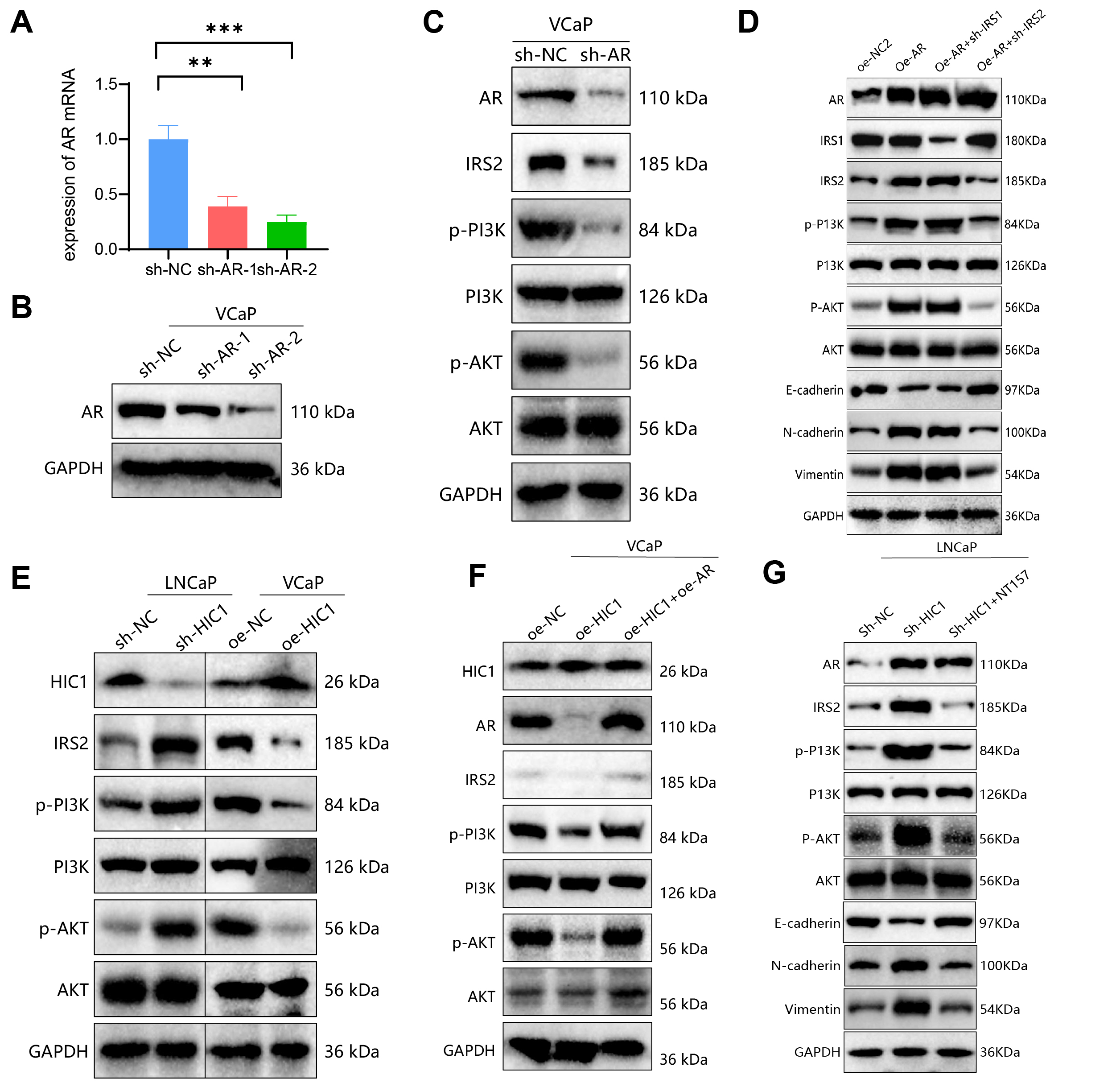

Supplement: Supplementary file 5 — Figure S4 [file CCS3-19-e12032-s004.jpg]

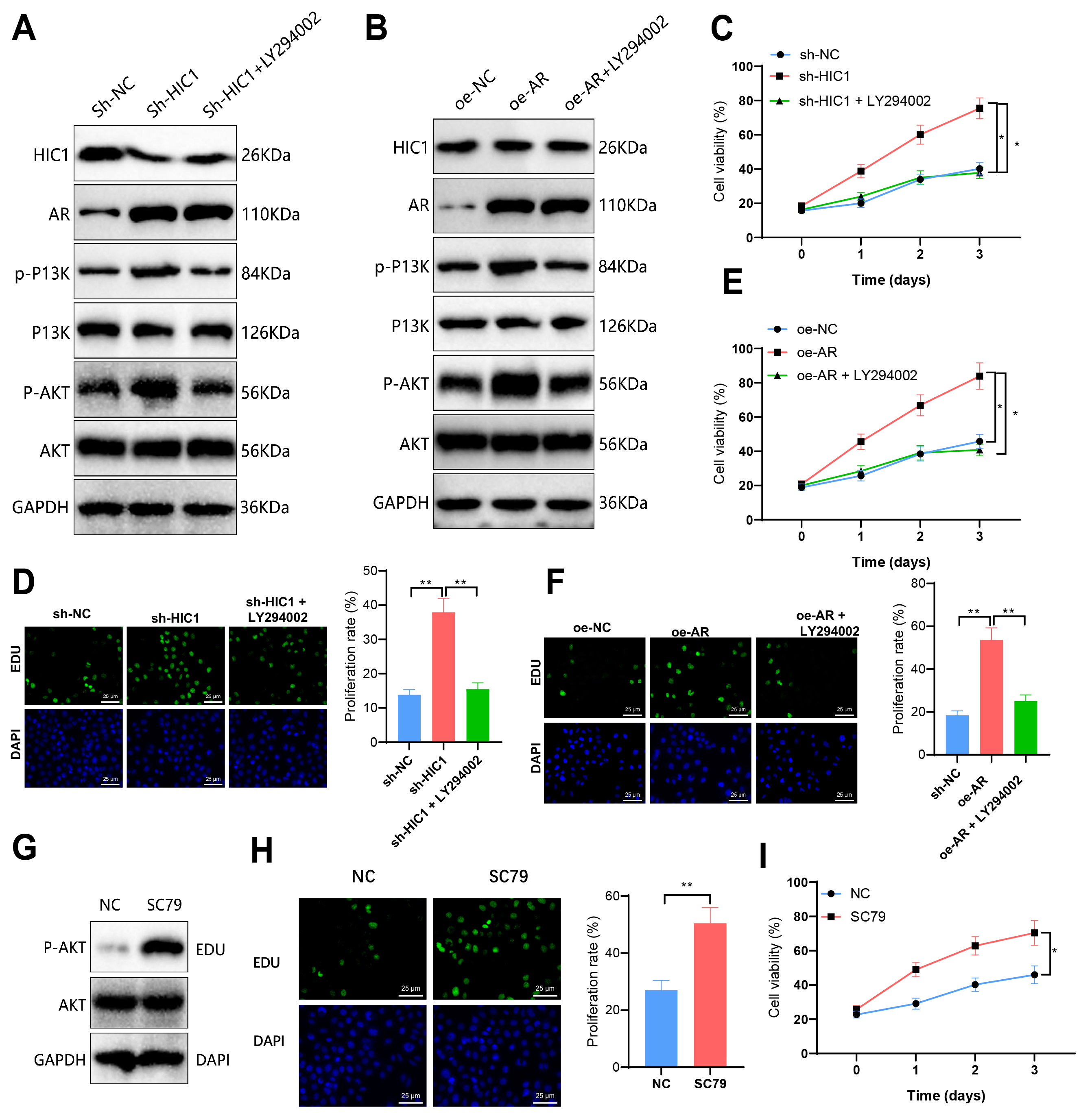

Supplement: Supplementary file 6 — Figure S5 [file CCS3-19-e12032-s002.jpg]

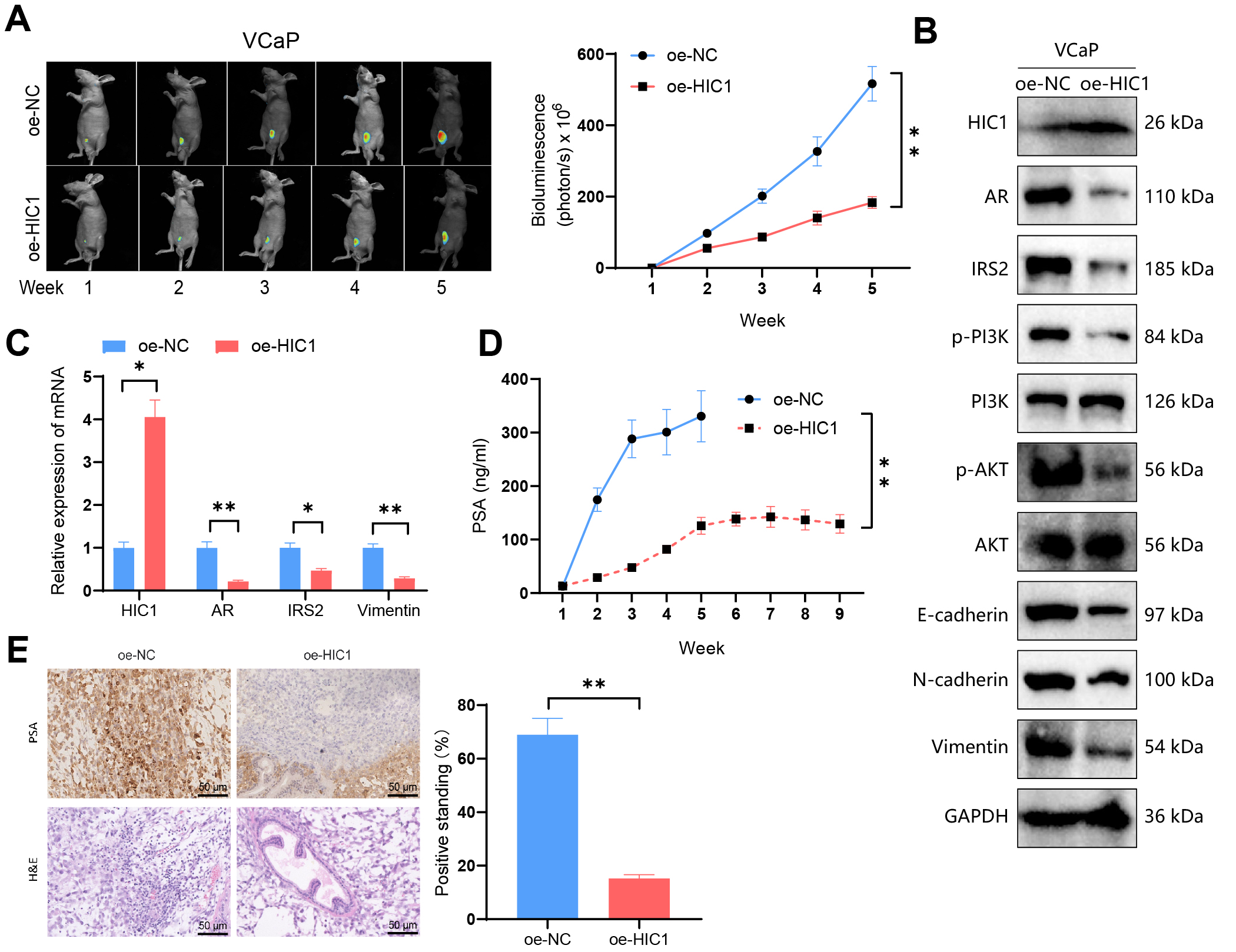

Supplement: Supplementary file 7 — Figure S6 [file CCS3-19-e12032-s007.jpg]
